# Thermotolerance evaluation of Taiwan Japonica type rice cultivars at the seedling stage

**DOI:** 10.1186/s40529-019-0277-7

**Published:** 2019-12-05

**Authors:** Ta-Ping Hsuan, Pei-Rong Jhuang, Wen-Chin Wu, Huu-Sheng Lur

**Affiliations:** 1 Hualien District Research and Extension Station, Council of Agriculture, Executive Yuan, Hualien, 97365 Taiwan (R.O.C.); 2 Department of Agronomy, National Taiwan University, Taipei, 10616 Taiwan (R.O.C.)

**Keywords:** Rice, Subtropical, Japonica type rice, High temperature, Seedling stage, Temperature induction response technique (TIR), Thermotolerance, Variety screening tool

## Abstract

**Background:**

The subtropical rice varieties grown in Taiwan are mainly Japonica-type rice varieties, which are grown in the southernmost- and lowest-latitude Japonica type rice cultivation area in the world. In Taiwan, seedlings that are planted either by transplanting or direct seeding in the second crop will face the season with the highest temperatures during the year. High-temperature stress severely influences early rice growth and causes yield losses. With global warming deteriorating, this problem is becoming increasingly severe. This study attempted to establish a high-efficiency and time-saving screening tool for rice varieties that exhibit thermotolerance during the early growth stages and further identify good donors with better tolerance for high temperature stress from Taiwan Japonica type rice germplasm.

**Results:**

During the initial germination stage, there were significantly different responses to heat stress among the different rice varieties. After the temperature induction response technique (TIR) treatment, the seedling survival rate and relative growth rate of the rice varieties under high temperature stress were significantly improved. In addition, the seedlings of the thermotolerant varieties demonstrated greater thermotolerance performance in the pot experiment as well as cell membrane stability (CMS) and cell activity (2,3,5-triphenyl-tetrazolium chloride; TTC) test results. However, the correlation between the thermotolerance of the seedlings and seeds was low. A phylogenetic dendrogram was plotted and revealed that thermotolerant genes did not concentrate in specific clusters. Furthermore, there was a non-significant correlation between the thermotolerance of the varieties and the years in which they were released.

**Conclusions:**

The temperature induction screening tool established by this study could determine the potential of each variety to adapt to high temperature stress. Additionally, thermotolerance during different growth stages (i.e., the germination, seedling, and grain maturation stages) exhibited low correlations. In this study, the varieties obtained through preliminary screening (i.e., TK14, HC56, TT30, TNG70, and TK8) exhibited outstanding thermotolerance. The screen tools and thermotolerance varieties could be valuable resources for the countries that grow Japonica type rice to apply when breeding thermotolerant varieties in the future.

## Background

According to predictions of the Intergovernmental Panel on Climate Change (IPCC), by the end of the twenty-first century, the average global temperature is expected to increase by 1.8–4 °C (IPCC 2007). Furthermore, in the next century, the average global temperature is predicted to increase again by 1.1–6.4 °C (IPCC 2012). Rice is most sensitive to heat during the flowering and early grain-filling stages. For example, rice exposed to a temperature of 33 °C for 4–6 h on flowering day results in more than 50% sterile rice grains, thereby severely influencing its production (Satake 1995). Furthermore, 1 °C increases in day and night temperatures reduce rice production rates by 7–8% (Baker et al. 1992) and 15% (Peng et al. 2004), respectively. In addition to the severe reduction in rice production, the increase in the proportion of chalky and fissured kernels under high temperature has seriously affected rice quality in Japan (Kobata et al. 2004; Nagata et al. 2004). Thus, the breeding of highly thermotolerant rice varieties is crucial for adapting to future global warming.

Rice is Taiwan’s main food crop, with two crops harvested during the year. The seedling process of the second crop is conducted from June to August when temperatures are highest. Therefore, high air and water temperatures pose obstacles to rice seedling growth and reduce the tiller number of grown rice (Oh-e et al. 2007), thus becoming the main reasons for the low production rate of Taiwan’s second rice crop (Lin and Chen 1976). Currently, rice cultivation in Taiwan mainly employs transplantation. In the face of a severe labor and irrigation water shortage and increasing labor costs in rural areas, Taiwan has attempted to replace the conventional transplanting method with direct seeding. However, the initial air and water temperatures pose substantial growth risks for Taiwan’s crops, which are commonly grown using wet direct-seeding. Direct seeding in ungraded fields may cause seeds to be exposed over the soil surface or sink into the ground surface. If met with high air and water temperatures, such seedlings will be injured, thereby causing irregular germination (Yang et al. 1997). Furthermore, shortages exist in thermotolerant varieties that are capable of handling direct seeding. Numerous studies have been conducted on the thermotolerance of rice varieties during the heading and grain-filling stages, including spikelet fertility changes under various temperatures and the screening of numerous high-tolerance varieties (Tenorio et al. 2013). However, insufficient studies have focused on evaluating rice varieties with thermotolerant seedlings. Under the threat of rising temperatures caused by global warming, efficient thermotolerant seedling screening methods could assist in the selection of thermotolerant rice varieties to adapt to global warming.

Yeh et al. (2012) proposed the concept of thermotolerance diversity, which divides thermotolerance into basal thermotolerance (BT), in which a plant demonstrates the ability to tolerate heat stress without prior exposure to heat acclimation, short-term thermotolerance (SAT) in which a plant demonstrates tolerance after hours of recovery from prior exposure to heat stress, long-term thermotolerance (LAT), in which a plant demonstrates tolerance after 2 to 3 days of recovery from prior exposure to heat stress, and thermotolerance to moderately high temperatures (TMHT), in which a plant demonstrates tolerance after prolonged exposure to moderately high temperatures. In addition, because different genes participate in different thermotolerance mechanisms, such mechanisms can withstand varying degrees of exposure and periods of high temperatures.

Seeds harvested in different climates and environments may exhibit varying degrees of thermotolerance (Fukushima et al. 2015; Permana et al. 2017). Kumar et al. (1999) stated that because thermotolerance performance is attributed to the genetic expression of diverse genes and heat shock protein mechanisms, the evaluation of plant thermotolerance properties should be conducted after the temperature induction process. Therefore, Kumar proposed the temperature induction response (TIR) technique to screen thermotolerant sunflower varieties. During the seedling stage, the seedlings were exposed to gradually increasing temperatures to induce the plant’s thermotolerance mechanism. Subsequently, the seedlings were subjected to extremely high temperature treatments to compare the thermotolerance performance. The TIR technique has been applied to screen thermotolerant peas (Srikanthbabu et al. 2002), cotton (Kheir et al. 2012), *Eleusine coracana* (Babu et al. 2013), and rice (Harihar et al. 2014; Vijayalakshmi et al. 2015), thereby demonstrating the TIR technique to be an effective screening method for acquiring thermotolerant varieties.

Taiwan consumes rice as its primary food crop, and Japonica type rice currently accounts for over 95% of Taiwan’s rice cultivation area. Taiwan is the southernmost- and lowest-latitude cultivation area for Japonica type rice (Lur 2009). Therefore, rice cultivation in Taiwan faces further severe effects from global warming. According to the prediction of Chou et al. (2017), in the worst-case scenario, Taiwan’s temperature may increase by 3.0–3.6 °C by the end of the century, with the annual average number of heatwave days increasing from the current 20 days to 110–180 days. In the future, extreme heatwave events will span the entire summer in Taiwan. Therefore, Taiwan’s second-crop rice seedlings face even harsher future challenges. In addition to the urgent need for thermotolerant varieties, time-saving and high-efficiency thermotolerance screening tools are crucial for evaluating the thermotolerance performance of each variety. Therefore, this study employed high temperature (HT) treatment and the TIR technique as screening methods to select rice varieties during the seedling growth stage. The feasibility of the proposed method for screening thermotolerant varieties during the seedling stage was also evaluated. This report evaluated the heat tolerance of 23 local important rice cultivars in Taiwan and applied the N22, Giza 178, and Koshiibuki varieties, which have been verified by international researches to exhibit thermotolerance during the grain-filling stage (Hoshi et al. 2001), and Koshihikari (the main cultivar of Japan), as the control variety. It was hoped that thermotolerant varieties at the seedling stage could be screened for future breeding.

## Materials and methods

### Preparing the rice seedlings

Surface sterilization of the seeds was conducted using a 1% sodium hypochlorite solution for 30 min. The seeds were placed in a water bath (G-20; Kingtech Scientific Co., Ltd., Taiwan) at 28 ± 2 °C to induce germination. When the seeds’ shoots had grown to 0.2 cm, the plants were wrapped in water-absorbent paper towel and placed onto a water plate. After the seedlings had grown to 1.5–2.0 cm, the following treatments were conducted.

### Establishing the high-temperature treatment conditions for rice seedlings

Seedlings with homogenous and normal phenotypes were selected and wrapped in paper rolls, and then subjected to water-bath treatments at 44, 46, 48, and 50 °C. Each treatment was repeated three times, with each repeated process involving six plants in each wrap. During the water-bath treatment, the paper-wrapped seedlings were soaked in the flow circulation of a temperature-controlled water bath for 3 h. Subsequently, the rice seedlings were placed in a temperature-controlled growth chamber at 30 °C. After 5 days, the seedling survival rate (%) and shoot relative growth rate (shoot RGR) were investigated. The shoot RGR (%) was calculated using the following equation: T/C × 100%, where C and T refer to the amount of growth of the shoot length in the control seedlings that did not receive heat treatment and those that received heat treatment, respectively. Growth was calculated according to the after-treatment shoot length minus the before-treatment shoot length.

### Screening thermotolerance of rice varieties

In this study, 27 cultivars were used (see Table 1). The seedlings were grown according to the aforementioned method with three replicates per treatment and six plants per replicate. The treatments included 30 °C for 3 h (control; CK); direct high temperature treatment (HT) at 45, 48, and 50 °C for 3 h (HT45, HT48, and HT50); and a progressive temperature induction performed first followed by high temperature treatments at 48 and 50 °C for 3 h (TIR48 and TIR50). The entire process, from applying the temperature induction technique to administering the high temperature treatment, was referred to as the TIR treatment in this study. Specifically, the TIR treatment entailed applying progressive temperature induction in a circulating temperature-controlled water bath device (G-20; Kingtech Scientific Co., Ltd., Taiwan) for 3 h prior to the HT treatment, which involved increasing the temperature from 35 to 42 °C over the course of 3 h. After the induction process, the seedlings were placed at room temperature to recover for 1 h before undergoing 48 °C and 50 °C HT treatments for 3 h. The rice plants’ survival rate (%) and the shoot RGR (%) were investigated at 5 days after treatment.

**Table 1:** List of the rice varieties used in the present study

Sub-species	Variety name	Abbreviation	Origin	Release year
Japonica	Chialungyu 242	CNG242	Taiwan	1956
	Hsinchu 56	HC56	Taiwan	1953
	Hualien 21	HL21	Taiwan	2008
	Kaohsiung 139	KH139	Taiwan	1975
	Kaohsiung 145	KH145	Taiwan	2004
	Koshihikari	Koshihikari	Japan	1956
	Koshiibuki	Koshiibuki	Japan	2003
	Taichung 65	TC65	Taiwan	1936
	Taichung 192	TC192	Taiwan	2007
	Taikeng 2	TK2	Taiwan	1989
	Taikeng 4	TK4	Taiwan	1990
	Taikeng 8	TK8	Taiwan	1992
	Taikeng 9	TK9	Taiwan	1993
	Taikeng 14	TK14	Taiwan	1996
	Taikeng 16	TK16	Taiwan	1997
	Taikeng 17	TK17	Taiwan	1998
	Tainan 5	TN5	Taiwan	1965
	Tainan 11	TN11	Taiwan	2004
	Tainung 67	TNG67	Taiwan	1978
	Tainung 70	TNG70	Taiwan	1985
	Tainung 71	TNG71	Taiwan	2000
	Taitung 30	TT30	Taiwan	2002
Indica	Giza 178	Giza 178	Philippine	1995
	Nagina 22	N22	India	1978
	Taichung Native 1	TN1	Taiwan	1960
	Taichung Sen10	TCS10	Taiwan	1979
	Taichung Sen 197	TCS197	Taiwan	2016

### Phylogenetic dendrogram

This study referenced Wu and Lin (2008) when drawing the phylogenetic tree. The coefficient of parentage refers to the estimated value of genetic similarities between varieties (Kempthorne 1969) and represents the chances of any allele on any genetic locus of two varieties being identical by descent. This coefficient was calculated in accordance with pedigree information and a computer program written by Lin and Wu (1994). The pedigree information in this study was established based on the database completed by Wu (2008), and the pedigree information of foreign varieties were added (e.g., Koshiibuki, Nagina 22, and Giza 178). The computer program was written using the Pascal programming language. The calculation program employed was similar to that in Chen et al. (2008). The algorithm logic adopted the hypotheses in Cox et al. (1986), which are described as follows: (1) each hybridization parent provides 50% of its genetic composition to the variety; (2) the primitive ancestors are unrelated to each other (coefficient of parentage = 0); (3) because rice is a self-pollinated crop, the primitive ancestors and hybridization parents are hypothesized to be homozygotes and homogeneous; and (4) the coefficient of parentage between the variety and direct parent is 0.75. In addition, the primitive ancestors in this study were defined as parents with untraceable pedigrees and hybridization records. The computer program calculated the coefficient of parentage between the 27 varieties used in this study. Cluster analysis was conducted using the UPGMA algorithm to determine the distance between each cluster, and the R software package was employed to produce a dendrogram.

### Influence of high temperature on rice seed germination

Sterilization and germination induction were conducted on the seeds of the 27 varieties listed in Table 1. First, ten rice seeds from each variety were selected and placed in a 9-cm petri dish that featured a filter paper. Subsequently, 15 mL of sterile water was poured into the petri dish. The petri dish was then placed in 30 °C (CK) and 42 °C growth chambers for 14 days. There were four replications in every treatment. After 5 days in the growth chamber, the rice seed germination rates were examined regularly until day 14. The germination rate calculation formula and statistical analysis method were as follows:

- Relative germination (%) = (GNt/GNck) × 100
  GNt: number of germinated seeds that underwent 42 °C heat treatment; GNck: number of germinated seeds that underwent 30 °C treatment
- LSD (α = 0.05) was employed to compare the germination rate between each treatment.

### Thermotolerance performance of thermo-tolerant and thermo-sensitive varieties after direct seeding into pots

This experiment selected four varieties that exhibited outstanding thermotolerance performance (TK14, TNG70, HC56, and TK8) and two species with thermosensitive performance (N22 and KH145) in terms of the results from the aforementioned screening method. After germination was induced, the seeds were planted into plastic pots that were 8.5 cm wide and 6.5 cm deep and which contained 150 mL of seed-starting mix. Each plastic pot was seeded with nine rice plants. During the growth and inspection period, the pots were placed in an incubator that maintained a temperature of 28 ± 2 °C and had 12 h of lighting daily. Treatments were administered when the seedlings’ shoots had grown to 1.5–2 cm. During this experiment, the temperature was 30 °C for CK, and the HT and TIR treatments were conducted for 3 h at 50 °C. Four pots were used for this experiment, yielding four trials in total. The rice seedlings’ survival rates and shoot RGR were measured before, immediately after, 5 days after, and 7 days after treatment.

### Measuring the relative injury (RI), cell activity (TTC), and malondialdehyde content (MDA) of thermotolerant rice seedlings

This experiment selected the seeds of four varieties that exhibited outstanding thermotolerance performance (TK14, TNG70, HC56, and TK8) and two varieties with poor thermotolerance performance (N22 and KH145) in terms of the results from the aforementioned screening method. The temperature was 30 °C for CK, and the HT and TIR treatments were conducted for 3 h at 50 °C.

### Cell membrane stability (CMS)

By modifying the treatment in Hsu and Yeh (2004), this study selected five healthy 1.5–2-cm seedling shoots from the wraps and removed the seed and roots. After being rinsed with distilled water, the seedlings were placed in test tubes with 15 mL of preheated distilled water. Each test tube contained five seedlings. Because this procedure was repeated once for each test tube, the procedure was repeated four times. After using water baths to administer the treatments, the test tubes were placed in a dark environment at 4 ± 1 °C for 24 h. Subsequently, the test tubes were removed from the dark environment and a conductivity meter (SC-2300; Suntex, Taiwan) was used to analyze the test tube content; the measured value was set as A. The test tubes were then placed in a high-pressure autoclave at 121 °C and a pressure of 1.2 kg/cm^2^ to kill the plant cells. After cooling, the conductivity meter was employed to measure the conductivity of the test tubes’ contents; the measured values were set as B. The electrical conductivity (EC) was calculated as A/B × 100, and the RI (%) was calculated as

$$\left[ { 1- \left( { 1{-}{\text{T}}} \right)/\left( { 1- {\text{C}}} \right) \times \, 100\% } \right],$$

where T and C refer to the treatment and the control EC value, respectively.

### TTC cell viability assay

To conduct a TCC cell viability assay, this study modified the method of Fokar et al. (1998). First, the fresh weight (0.02 g) of the rice seedling tissue was recorded after it had been washed. Then, the seedling tissue was thoroughly soaked in 2 mL of 0.8% TTC solution in darkness for 24 h. The reddened root was extracted by 1.5 mL of 95% alcohol for 24 h. After the red substance had dissolved into the alcohol, the plant was removed, and the alcohol solution was fixed to a quantity of 1.5 mL. After evenly shaking the solution, a spectrophotometer was employed to determine the solution’s absorbance value (OD) at 530 nm. The root activity calculation was TTC = OD/tissue fresh weight (g), and the elative activity calculation was TTC (%) = T/C × 100%, where T and C refer to the treatment and control TTC values, respectively.

### MDA content analysis

Referring to the experimental process of Kao (2005), this study obtained 0.1 g of tissue from the rice shoots and roots and ground it equally in 2 mL of TCA. The tissues then underwent centrifugation at 10,000*g* for 5 min. Subsequently, 1 mL of supernatant was extracted from the solution and placed in a test tube, and then 4 mL of TCA was added. The test tube was heated in a 95 °C hot water bath for 30 min before quickly being inserted into ice to halt the reaction. Subsequently, the test tube underwent centrifugation at 3000*g* for 10 min to remove air bubbles.

Measurement method: A spectrophotometer was employed to measure the absorbance values of A_532_ and A_600_. This study used 1 mL of TCA (5%) to replace the extraction solution as the control group. The equation used was as follows:

$$\begin{aligned} {\text{MDA }}\left( {{\text{nmol}}\;{\text{g}}^{ - 1} } \right) & = \left( {{\text{A}}_{ 5 3 2} - {\text{A}}_{ 600} } \right)/ 1 5 5 { }\left( {{\text{K}},{\text{mM}}^{ - 1} \;{\text{cm}}^{ - 1} } \right) \times 5\,\left( {\text{reaction volume}} \right) \\ & \quad \times 4\,\left( {\text{dilution factor}} \right) \times 1000/{\text{FW }}\left( {\text{g}} \right). \\ \end{aligned}$$

$${\text{Relative MDA content}} = {\text{Treatment MDA content}}/{\text{CK MDA content}}.$$

## Results

### Evaluating the high-temperature thresholds of rice lethality and establishing high temperature treatment conditions

To determine the survival rates and lethal temperatures of the freshly germinated rice seedlings, various temperature (i.e., 44, 45, 46, and 48 °C) and time (i.e., between 1 and 3 h) conditions were separately employed in this study to evaluate the high-temperature lethal thresholds of the rice seedlings. The results indicated that the survival rate of the seedlings gradually decreased with the increase of temperature and treatment time (Table 2). When the treatment time was under 3 h at 44 °C and 46 °C, the seedling survival rate of each variety was 66.7–100% and 0–25%, respectively. When the treatment temperature was increased to 48 °C for 3 h, only TCS10 survived. Therefore, the initial observation results indicated that 48 °C for 3 h was the lethal temperature for the rice seedlings.

**Table 2:** Survival rates of rice varieties under different treatment times and temperatures (%)

Temp. (°C)	Duration of temperature treatment (h)											
	Koshiibuki			N22			TCS10			TK9		
	1	2	3	1	2	3	1	2	3	1	2	3
44	100.00	100.00	100.00	91.70	75.00	66.70	100.00	83.33	83.33	100.00	83.33	83.33
45	100.00	100.00	34.40	77.80	50.00	16.70	100.00	100.00	80.16	83.33	33.33	5.53
46	60.00	50.0	25.00	38.90	11.10	0.00	66.67	50.00	16.67	33.33	33.33	0.00
48	17.10	6.30	0.00	0.00	0.00	0.00	18.75	16.67	15.83	16.67	0.00	0.00
Temp. (°C)	***			***			***			***		
Duration (h)	***			***			***			***		
T × hr	***			***			*			***		

ns, *, **, *** Means not significant, significant at P ≦ 0.05, 0.01 and 0.001, respectively

### Establishing screening methods for thermotolerance in rice seedlings and the evaluation of thermotolerant performance among Taiwan rice varieties

The screening results under various treatment conditions are presented in Tables 3 and 4. There were significant differences in the survival rate among rice varieties under the HT45 treatment. The average survival rate of each species was 34.2%. The survival rates ranged from 5.6 to 81.67%, indicating that the survival rates of the different rice varieties exhibited substantial differences, and that HT45 was suitable for screening thermotolerant rice varieties. Under the HT45 treatment, TN1 and TCS10 demonstrated outstanding survival rates, which were followed by those of HC56, TN5, TK2, and TC192. When the evaluation temperature exceeded the lethal temperature (48 °C), the seedling survival rate and shoot RGR (Tables 3 and 4) of all varieties were significantly reduced. Under the HT48 and HT50 treatments, the average survival rate was reduced to 9.3% and 9.7%, respectively. Compared with other varieties, TT30, Koshihikari, HC56, TN5, TN11, TK14, TK17, and HL21 performed more favorably under the HT48 treatment, whereas TT30, TK14, TK16, TK9, HL21, TK4, and TN11 performed more favorably under the HT50 treatment. Furthermore, a comprehensive evaluation of the HT45, HT48, and HT50 treatments indicated that TK14, HC56, TN11, TT30, and HL21 exhibited higher overall performance than did other species. However, if progressive temperature induction was administered before the 48 °C and 50 °C treatments (i.e., TIR48 and TIR50), the survival rate of all rice varieties improved greatly. Because most species exhibited survival rates greater than 90% under TIR48, this approach could not evaluate and compare the thermotolerance performance of the varieties using the survival rate. Therefore, the temperature was increased to 50 °C, at which the varieties demonstrated significantly different survival rates. The average seedling survival rate under TIR50 was 48.3% with a distribution range of 15.0–92.5%. After progressive temperature induction, the varieties that exhibited favorable thermotolerances included TNG70, TK2, TK14, TT30, TK4, TK17, HC56, and HL21. By contrast, N22 and Koshiibuki demonstrated inferior performance.

**Table 3:** Comparison of the seedling survival rates (%) of each rice variety under various high temperature treatments

Variety	HT			TIR	
	45 °C	48 °C	50 °C	48 °C	50 °C
CNG242	16.67 hijkl^a^	6.25 de	10.00 cdefg	100.00 a	36.67 gijk
HC56	59.17 b	20.83 bcd	7.50 efg	100.00 a	57.50 de
HL21	16.67 hijkl	16.66 bcde	20.00 bc	100.00 a	57.50 de
KH139	13.19 jklm	4.17 de	5.00 fg	100.00 a	32.50 ijk
KH145	22.22 ghijkl	4.17 de	12.50 cdef	83.33 c	46.67 fghij
Koshihikari	37.50 cdef	25.00 abcd	2.50 g	100.00 a	42.50 fghij
Koshiibuki	34.09 defgh	0.00 e	2.50 g	93.75 a	25.00 kl
TC65	37.50 cdefg	8.33 de	5.00 fg	97.92 a	42.00 ghij
TC192	50.00 bcd	5.56 de	5.00 fg	100.00 a	42.50 fghij
TK2	50.00 bcd	0.00 e	0.00 g	100.00 a	75.00 b
TK4	20.83 hijkl	4.16 de	15.00 bcde	95.83 a	60.00 cd
TK8	25.00 ghijk	11.11 cde	5.00 fg	100.00 a	52.50 defg
TK9	5.56 lm	0.00 e	20.00 bcd	100.00 a	55.00 def
TK14	16.67 hijkl	16.67 bcde	22.50 bcd	100.00 a	72.50 bc
TK16	30.83 efghi	5.56 de	22.50 b	100.00 a	45.00 efghi
TK17	34.09 defgh	16.67 bcde	5.00 fg	100.00 a	57.50 de
TN1	81.67 a	0.00 e	2.50 g	100.00 a	40.00 ghij
TN5	51.79 bc	20.83 bcd	10.00 cdefg	95.83 a	37.50 hijk
TN11	36.11 cdefgh	17.50 bcde	15.00 bcde	91.67 abc	52.50 defg
TNG67	28.12 fghij	0.00 e	2.50 g	100.00 a	46.67 defgh
TNG70	33.33 defgh	4.17 de	10.00 cdefg	91.67 abc	92.50 a
TNG71	15.97 ijklm	5.56 de	3.33 fg	95.83 a	30.00 jkl
TT30	45.83 bcde	25.00 a	35.00 a	91.67 abc	65.00 de
Giza 178	40.83 cdef	8.33 de	0.00 g	100.00 a	40.00 ghij
N22	12.50 klm	0.00 e	10.00 defg	85.00 bc	15.00 l
TCS10	80.16 a	15.42 cde	0.00 g	97.20 ab	47.50 defgh
TCS197	27.78 fghijk	8.33 de	12.50 cdef	93.33 ab	37.50 hijk
Mean	34.20	9.30	9.70	96.80	48.30
C.V.	0.59	1.12	1.05	0.07	0.37

^a^Means separation within columns by Fisher’s LSD test at *P *< 0.05

**Table 4:** Influence of HT and TIR treatments on the shoot RGR (%) of the rice varieties

Variety	HT		TIR	
	48 °C	50 °C	48 °C	50 °C
CNG242	5.09 bc^a^	4.83 abc	37.94 defg	16.79 abcde
HC56	14.49 a	3.33 bc	87.14 a	14.97 abcdef
HL21	0.77 c	6.39 abc	29.80 fg	21.54 a
KH139	4.50 bc	4.61 abc	63.09 bc	14.86 abcdef
KH145	5.03 bc	3.60 bc	21.71 g	4.89 ij
Koshihikari	6.21 bc	3.92 bc	53.76 bcde	9.28 efghi
Koshiibuki	2.41 c	2.38 c	39.57 cdefg	5.74 hi
TC65	4.07 bc	2.36 c	31.68 efg	10.77 defghi
TC192	17.05 a	3.10 bc	41.80 cdefg	10.95 defghi
TK2	3.04 bc	5.12 abc	20.80 g	16.06 abcdef
TK4	1.79 c	4.02 abc	22.59 g	7.18 hi
TK8	6.35 bc	4.99 abc	50.97 bcdef	20.57 ab
TK9	2.18 c	3.10 bc	45.96 bcdefg	11.93 cdefgh
TK14	7.72 bc	3.43 bc	83.42 ab	16.55 abcde
TK16	0.00 c	4.05 abc	71.87 ab	13.84 bcdefg
TK17	1.95 c	4.23 abc	40.93 cdefg	7.83 fghi
TN5	6.32 bc	3.96 abc	28.92 fg	7.18 ghi
TN11	4.57 bc	6.06 abc	51.89 bcde	12.13 cdefgh
TNG67	1.26 c	4.50 abc	60.71 bcd	17.33 abcd
TNG70	1.72 c	3.00 c	51.31 bcdef	19.19 abc
TNG71	8.12 abc	7.12 ab	53.33 bcde	10.95 cdefghi
TT30	4.50 bc	8.95 a	59.88 bcd	11.44 cdefghi
Giza 178	3.92 bc	3.52 bc	47.77 bcdefg	8.79 fghi
N22	2.24 c	3.23 bc	13.61 g	7.78 fghi
TCS10	3.67 bc	2.18 c	28.77 fg	10.16 defghi
TCS197	11.19 ab	3.80 bc	78.74 ab	8.22 fghi
TN1	3.19 bc	3.43 bc	25.08 fg	8.85 fghi
Mean	4.94	4.19	46	12.1
C.V.	0.81	0.36	0.43	0.38

^a^Means separation within columns by Fisher’s LSD test at *P *< 0.05

Under the TIR48 and TIR50 treatments, the rice seedlings’ growth was constrained despite maintaining a certain survival rate. This study employed the shoot RGR to further evaluate the thermotolerance of each species (Table 4). Under the HT48 and HT50 treatments, the average shoot RGR values of the rice varieties were 4.19% and 4.94%, respectively, indicating that the shoot growth was severely inhibited. The shoot RGR distribution under the HT48 treatment ranged from 0 to 17.05%, with TC192, HC56, TCS197, and TNG71 exhibiting greater performance. However, the shoot RGR values of the varieties that had demonstrated greater performance differed slightly with those of the other species. The differences in shoot RGR between each variety were even smaller under the HT50 treatment, ranging from 2.18 to 8.95%, with TT30, TNG71, and HL21 demonstrating slightly greater performance than the other varieties. Therefore, the TIR technique significantly improved the shoot RGR of the rice seedlings under high-temperature stress. Under the TIR48 treatment, the average shoot RGR was 46.0% and the distribution ranged from 13.61 to 87.14%; HC56, TK14, TCS197, TK16, KH139, TNG67, and TT30 demonstrated greater performance, whereas N22, TK2, KH145, TK4, and TN1 exhibited inferior performance. By contrast, after the TIR50 treatment, the shoot RGR of each variety was severely constrained, exhibiting an average shoot RGR of 12.1%. Additionally, the shoot RGR ranged from 4.89 to 21.54%, demonstrating smaller differences in results between varieties compared with those of TIR48. Under the TIR50 treatment, HL21, TK8, TNG70, TNG67, CNG242, TK14, and TK2 demonstrated relatively greater performance, whereas KH145, Koshiibuki, TN5, TK4, N22, and TK17 exhibited inferior performance.

**Fig. 1:**
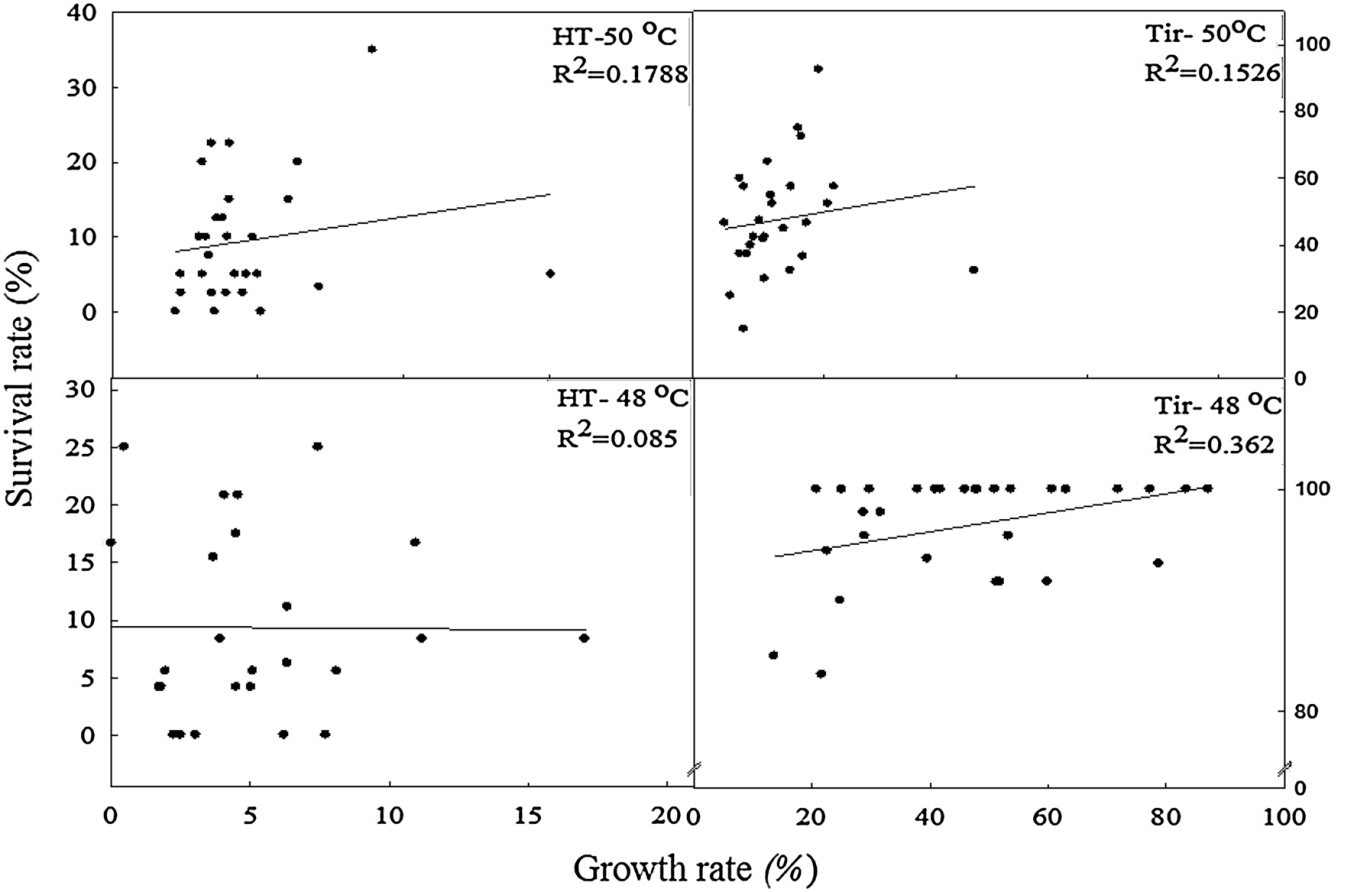
Correlation between seedling survival rate and shoot RGR under different HT and TIR treatments. Dots in the figure indicate varieties tested in this study and there are 27 varieties

Figure 1 further compares the relationship between the seedling survival rates and shoot RGR values under HT48, HT50, TIR48, and TIR50. The coefficient of determination (R^2^) values obtained from HT48, HT50, TIR48, and TIR50 were 0.085, 0.1788, 0.362, and 0.1526, respectively, indicating that the seedling survival rate and shoot RGR was not significantly correlated.

After sorting the results in Tables 3 and 4, this study employed the seedling survival rate and shoot RGR as screening indicators to select varieties that were thermotolerant and thermosensitive under different temperatures for the HT and TIR treatments (Table 5). Each variety reacted differently under different treatments, and they were grouped according to their heat stress responses as follows: (1) species that demonstrated greater thermotolerance under both the HT and TIR treatments (i.e., HC56, TK14, and TT30); (2) species that performed poorly under the HT treatment but favorably under the TIR treatment (for example, TNG70 performed poorly under the HT48 and HT50 treatments but demonstrated an outstanding seedling survival rate under the TIR50 treatment and excellent shoot RGR under the TIR48 and TIR50 treatments); and (3) species that displayed outstanding performance at lower temperatures but sharp performance declines when the temperature exceeded a certain threshold (for example, TCS10 exhibited a high seedling survival rate under the HT45 treatment but became thermosensitive under the HT48 and TIR48 treatments). In addition, N22 exhibited high heat sensitivity regardless of treatment. After a comprehensive comparison of the seedling survival rate and shoot RGR performances of each variety under the HT and TIR treatments, TK14, HC56, TT30, TK8, TNG70, and HL21 were selected in this study as thermotolerant species and N22, KH145, and Koshiibuki as thermosensitive species.

**Table 5:**
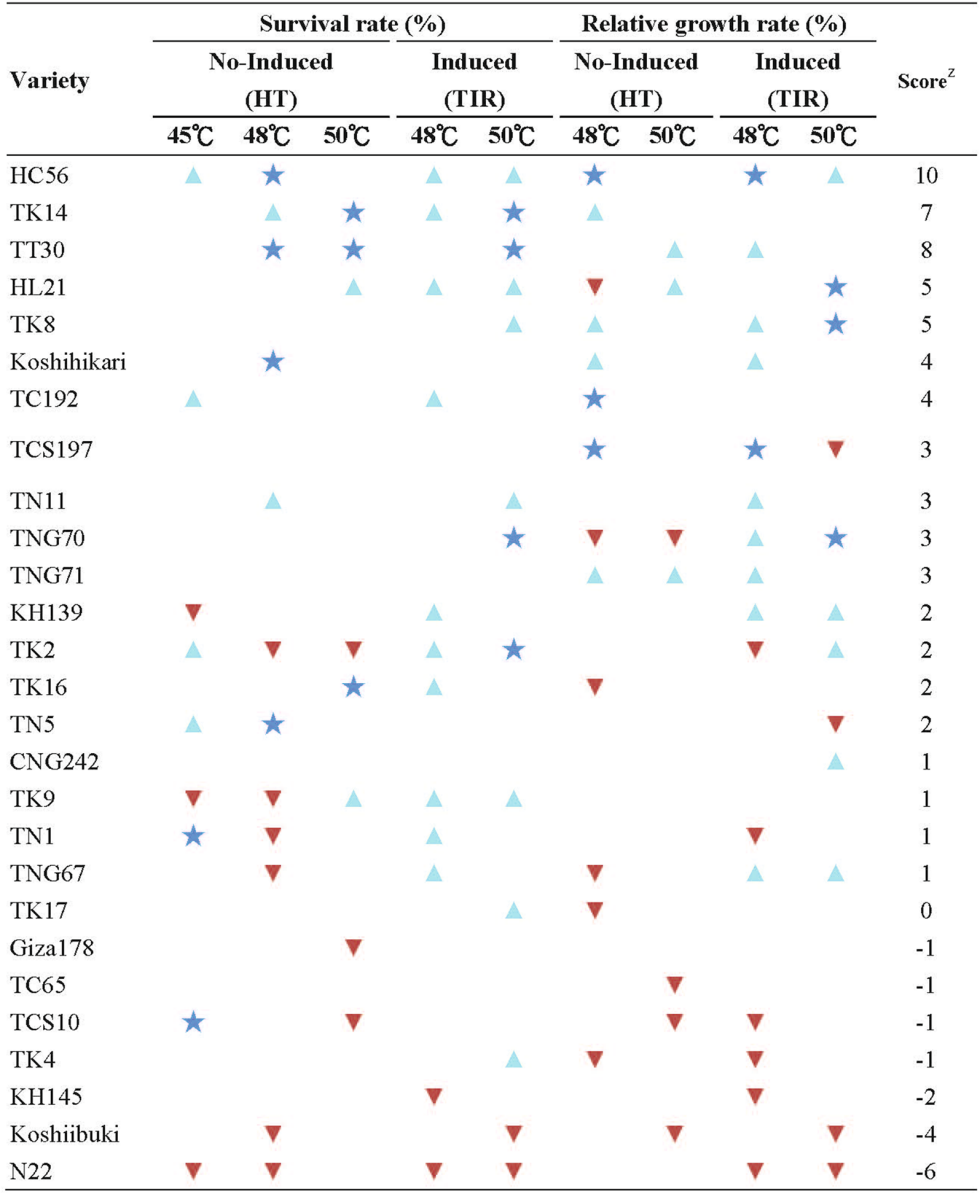
Thermotolerant and thermosensitive varieties screened according to various HT methods and indicators

: exhibited thermosensitivity,  : exhibited thermotolerance,  : exhibited high thermotolerance

^a^Means the score of each variety: According to the performance of the heat tolerance, each variety was separately rated as + 2, + 1 or − 1 to indicate high thermotolerance, thermotolerance or thermosensitivity in each treatment, and the varieties order in table was sorted according to the total score of each variety

### Relationship between the seedling thermotolerance of Taiwan rice varieties and their genetic relationship, release year, and seed germination rate

**Fig. 2:**
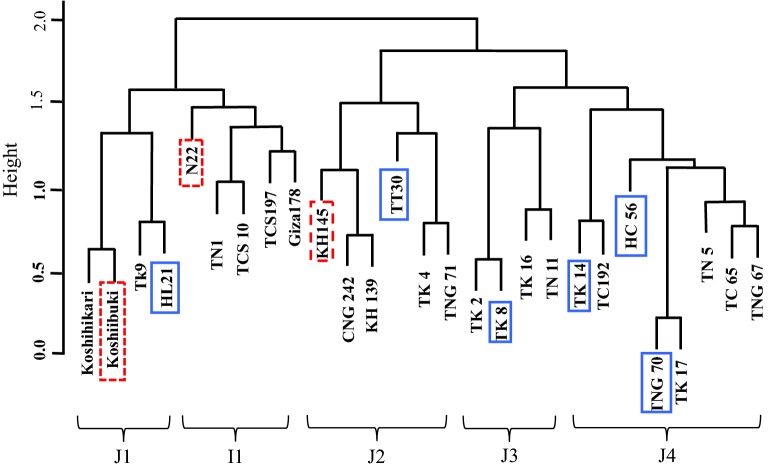
Distribution of rice varieties on the phylogenetic dendrogram. The tolerant and sensitive varieties in the tree are labeled as below:  : exhibited thermosensitivity,  : exhibited high thermotolerance

A phylogenetic dendrogram was plotted in this study for the 27 varieties according to their phylogenetic distances. Figure 2 indicates that the Indica-type varieties consisted of their own group (I1), while the Japonica-type rice varieties could be divided into four groups (J1, J2, J3, and J4).

By observing the distribution of thermotolerant varieties such as TK14, HC56, TT30, TK8, TNG70 and HL21 that were screened from the above comprehensive comparison on the phylogenetic dendrogram, the possible distribution of thermotolerance genes in these varieties could be discussed (Fig. 2). The results indicated that thermotolerant genes were not concentrated in specific clusters but instead scattered across all clusters. Furthermore, comparing the thermotolerance performance between Indica and Japonica type varieties (Fig. 2) revealed that Indica type varieties did not demonstrate significantly greater thermotolerance.

**Fig. 3:**
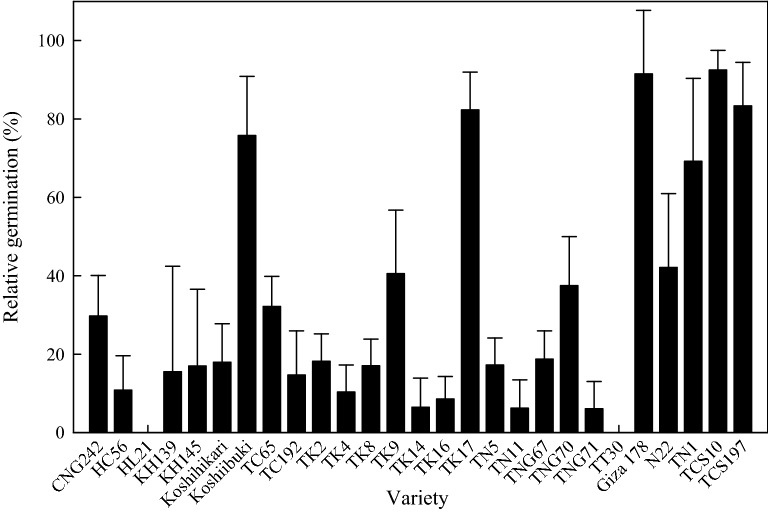
Relative germination rates of different varieties after 2 weeks of 42 °C treatment. Different letters above the bars indicate a significant difference at the 0.05 level

This study also explored the changes in germination rate under high temperature, and its correlation with seedling survival rate and shoot RGR. The relative germination rate in the 42 °C results showed that TCS10, Giza 178, TCS197, TK17, Koshiibuki, and TN1 demonstrated greater relative germination rates, whereas HL21 and TT30 demonstrated the lowest relative germination rates (Fig. 3).

**Fig. 4:**
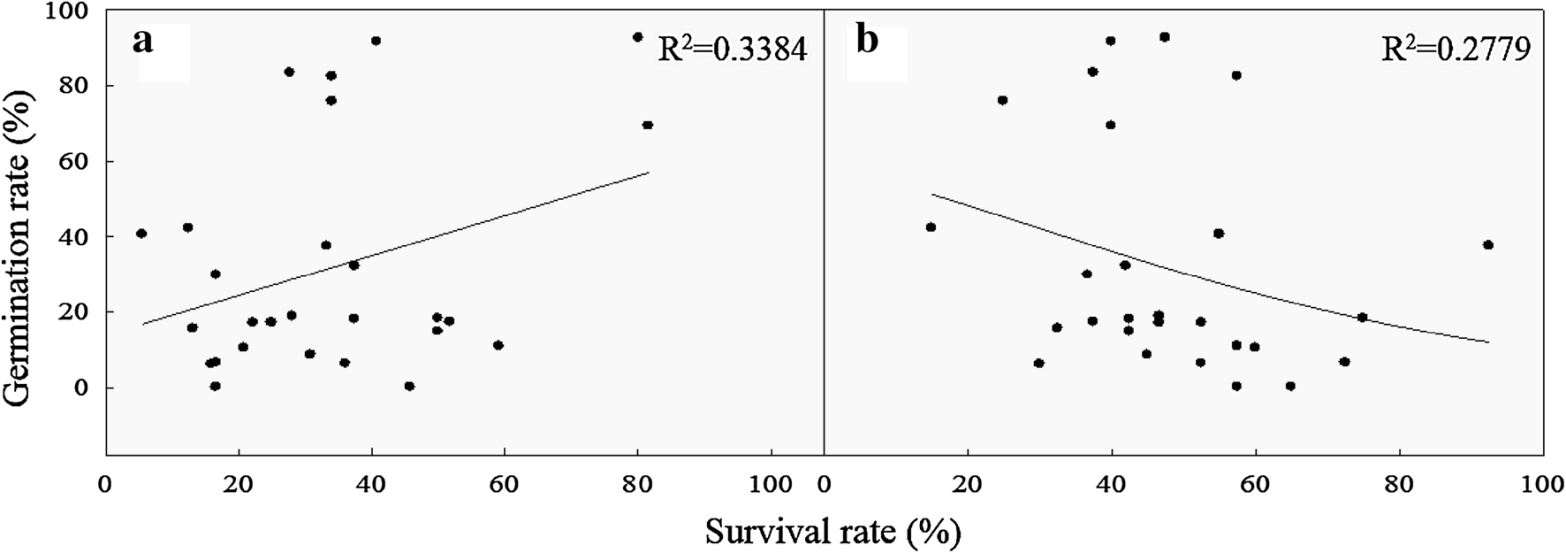
Correlation between survival and relative germination rates under the HT45 (**a**) and TIR50 (**b**) treatments. Dots in the figure indicate varieties tested in this study and there are 27 varieties

**Fig. 5:**
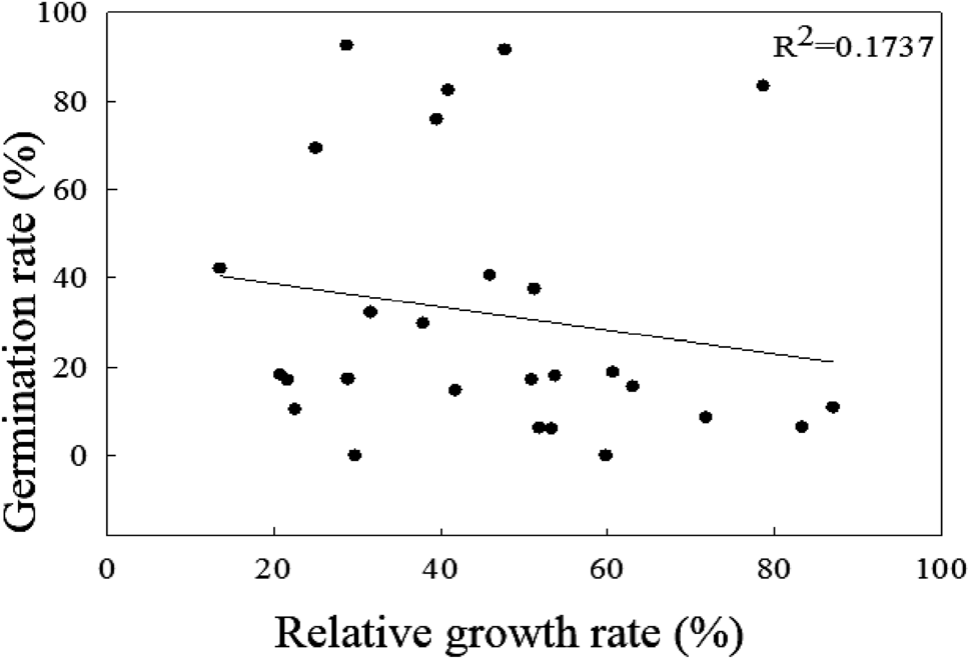
Correlation between shoot RGR and relative germination rate under the TIR48 treatment. Dots in the figure indicate varieties tested in this study and there are 27 varieties

Figures 4 and 5 present the correlations of the relative germination rates under the 42 °C treatment with the seedling survival rates under the HT45 treatment, the seedling survival rates under the TIR50 treatment, and the shoot RGR values under the TIR48 treatment, and demonstrate R^2^ values of 0.3384, 0.2779, and 0.1737, respectively. The results indicated that the relative germination rate under the 42 °C treatment was slightly correlated with the survival rate under HT45, the survival rate under TIR50, and the shoot RGR under TIR48.

**Fig. 6:**
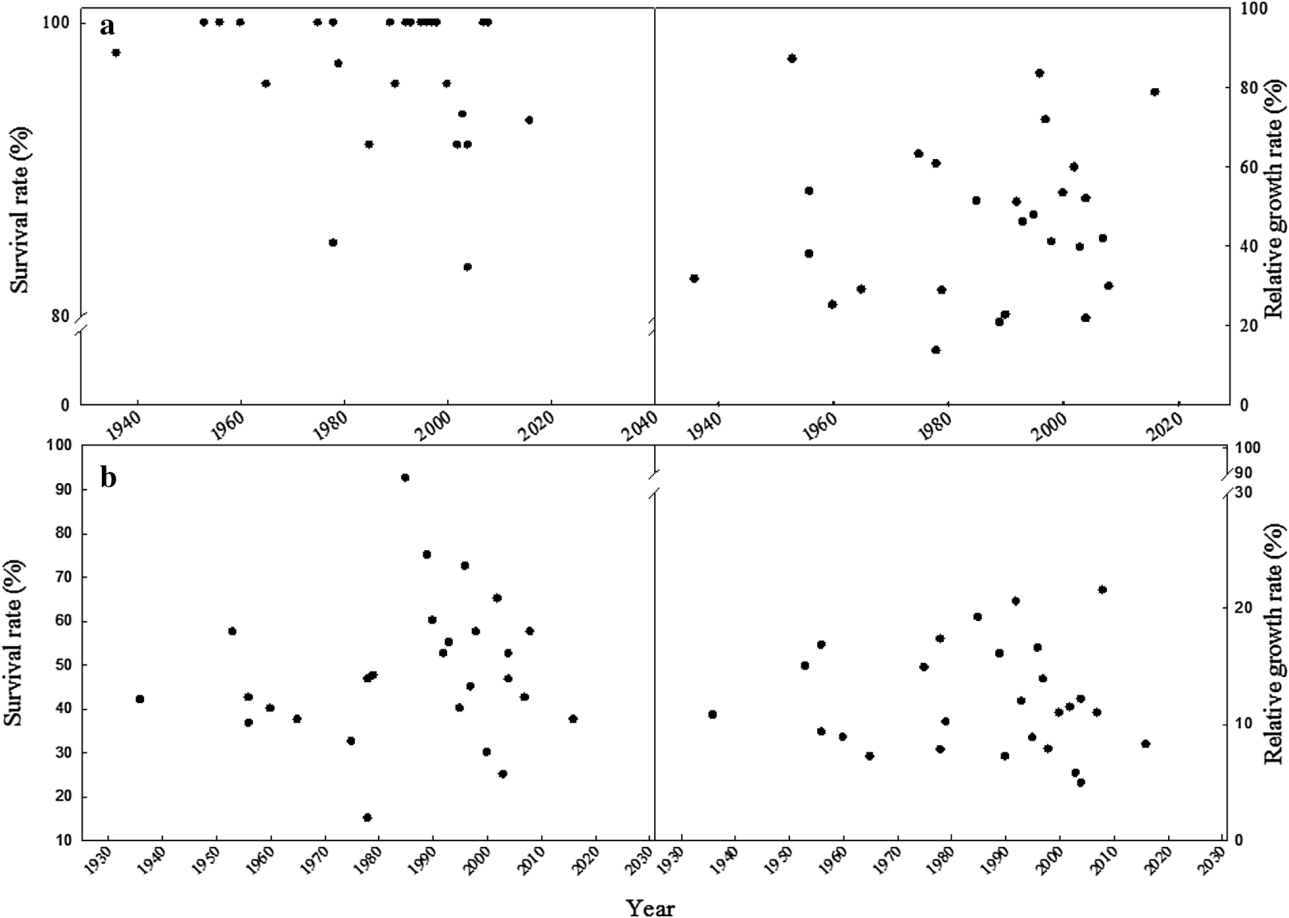
Correlation of seedling survival rates and shoot relative growth rate (RGR) with years that varieties were released under the TIR48 (**a**) and TIR50 (**b**) treatment. Dots in the figure indicate varieties tested in this study and there are 27 varieties

To discuss whether a correlation existed between varieties being bred under an environment of rising temperatures caused by global warming and their thermotolerance properties, this study plotted a distribution map of the seedling survival rates and shoot RGR values of all varieties under the TIR48 and TIR50 treatments in accordance with their release year (Fig. 6a, b). The results showed that under these treatments, the varieties demonstrating outstanding thermotolerance performances were distributed across multiple years, which indicated that thermotolerance was not related to the year that a variety was bred.

### Comparison of the properties of thermotolerant and thermosensitive varieties during the seedling stage

**Fig. 7:**
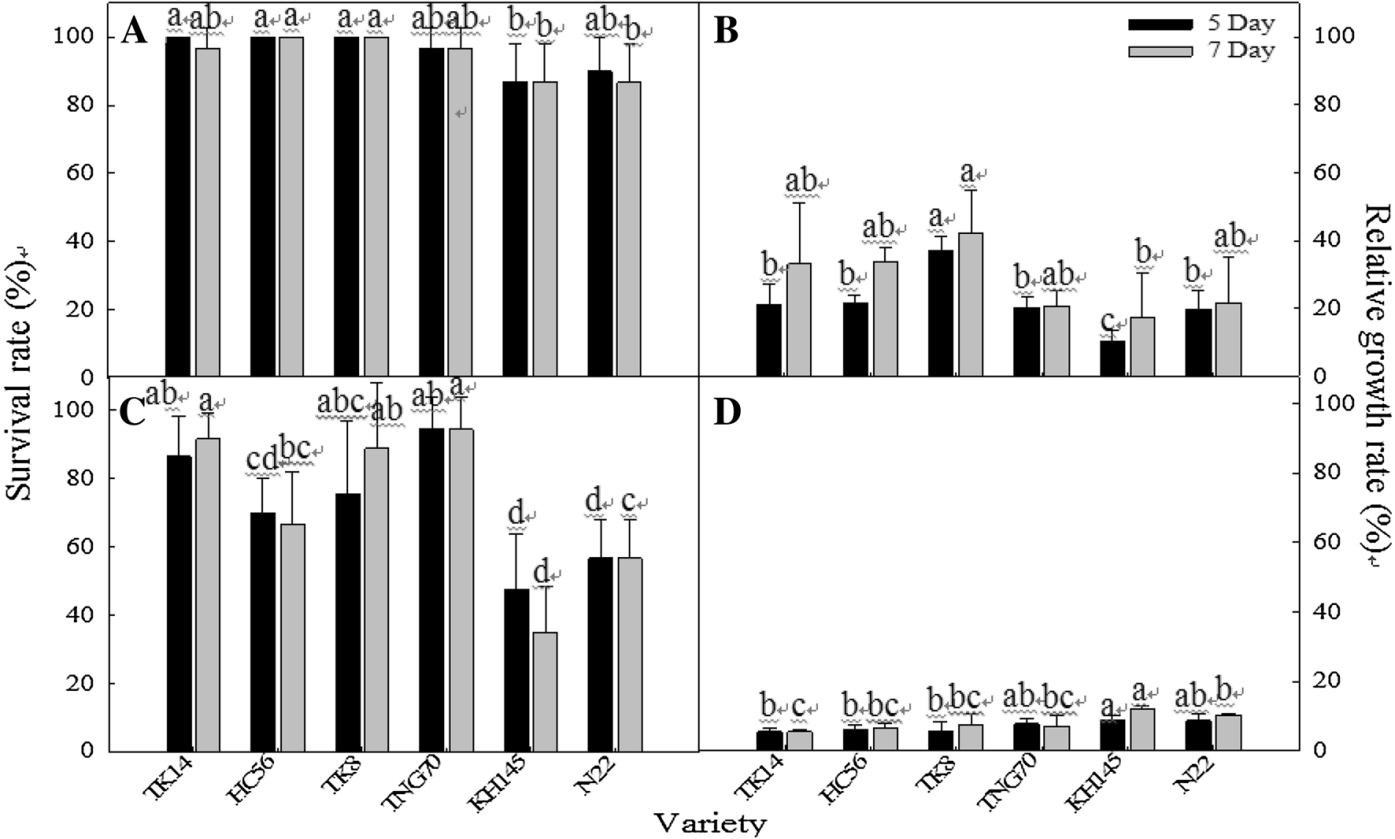
Seedling survival rate and shoot RGR performance of pot-cultivated rice 5 and 7 days after undergoing the TIR48 (**A**, **B**) and TIR50 (**C**, **D**) treatments. Different letters above the same color bars indicate a significant difference at the 0.05 level

The direct-seeding and pot cultivation experiment employed four thermotolerant varieties (TK14, TNG70, HC56, and TK8) and two thermosensitive varieties (N22 and KH145) (Fig. 7). The results indicated that the varieties exhibited significantly different shoot RGR values under the TIR48 treatments (Fig. 7B) and significantly different seedling survival rates under the TIR50 treatment (Fig. 7C). Furthermore, 5 days after the TIR48 treatment, TK8 demonstrated the highest shoot RGR values, whereas KH145 exhibited the lowest. Seven days after the TIR48 treatment, the shoot RGR of each variety substantially increased, with TK8 exhibiting the highest values and KH145 exhibiting the lowest (Fig. 7B). Moreover, under the TIR50 treatment, TNG70 and TK14 demonstrated higher seedling survival rates, whereas thermosensitive species (i.e., N22 and KH145) exhibited lower rates (Fig. 7C).

In sum, the seedling survival rate and shoot RGR performances of the thermotolerant and thermosensitive rice varieties following the TIR48 and TIR50 treatments demonstrated that the varieties screened using the wrapping method exhibited similar thermotolerant performances when directly seeded into pots. However, the shoot RGR results indicated that the thermotolerance levels of the screened varieties were lower when grown using the direct-seeding pot-cultivation method than when grown using the wrapping method (Table 4 and Fig. 7). The pot-cultivation survival rates were better than those for the wrapping method (Table 3 and Fig. 7).

### Physiological property analysis of thermotolerant and thermo-sensitive rice varieties in terms of CMS, TTC and MDA

**Fig. 8:**
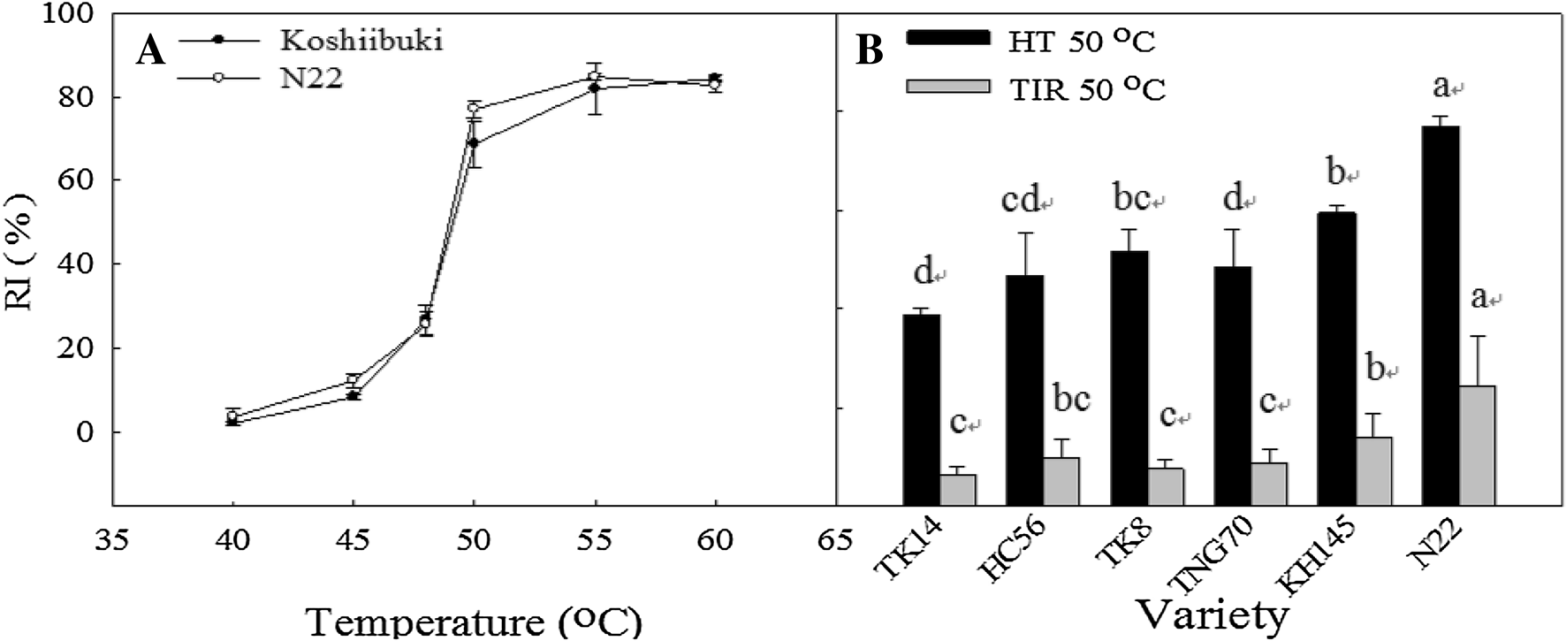
Relative injury (RI) values of rice seedlings under different temperature treatments (**a**) and the effect of TIR50 and HT50 on the RI (%) of rice seedlings (**b**); different letters above the same color bars indicate a significant difference at the 0.05 level

To understand the CMS performance of each variety under different high temperatures, this study first measured the EC values of Koshiibuki and N22 between temperatures of 40 to 60 °C and converted them to RI values, which increased substantially under the 50 °C treatment (Fig. 8a). Therefore, this study employed 50 °C as the temperature to measure the CMS performance of the four thermotolerant varieties and two thermosensitive varieties screened previously. The results indicated that under both the HT50 and TIR50 treatments, the RI (%) of the thermotolerant varieties (i.e., TK14, HC56, TK8, and TNG70) were significantly lower than those of the thermosensitive varieties (N22 and KH145) (Fig. 8b). This indicated that the thermotolerant varieties exhibited greater CMS than did the thermosensitive species, and that performing the TIR technique before the HT treatment improved the CMS of each variety.

**Fig. 9:**
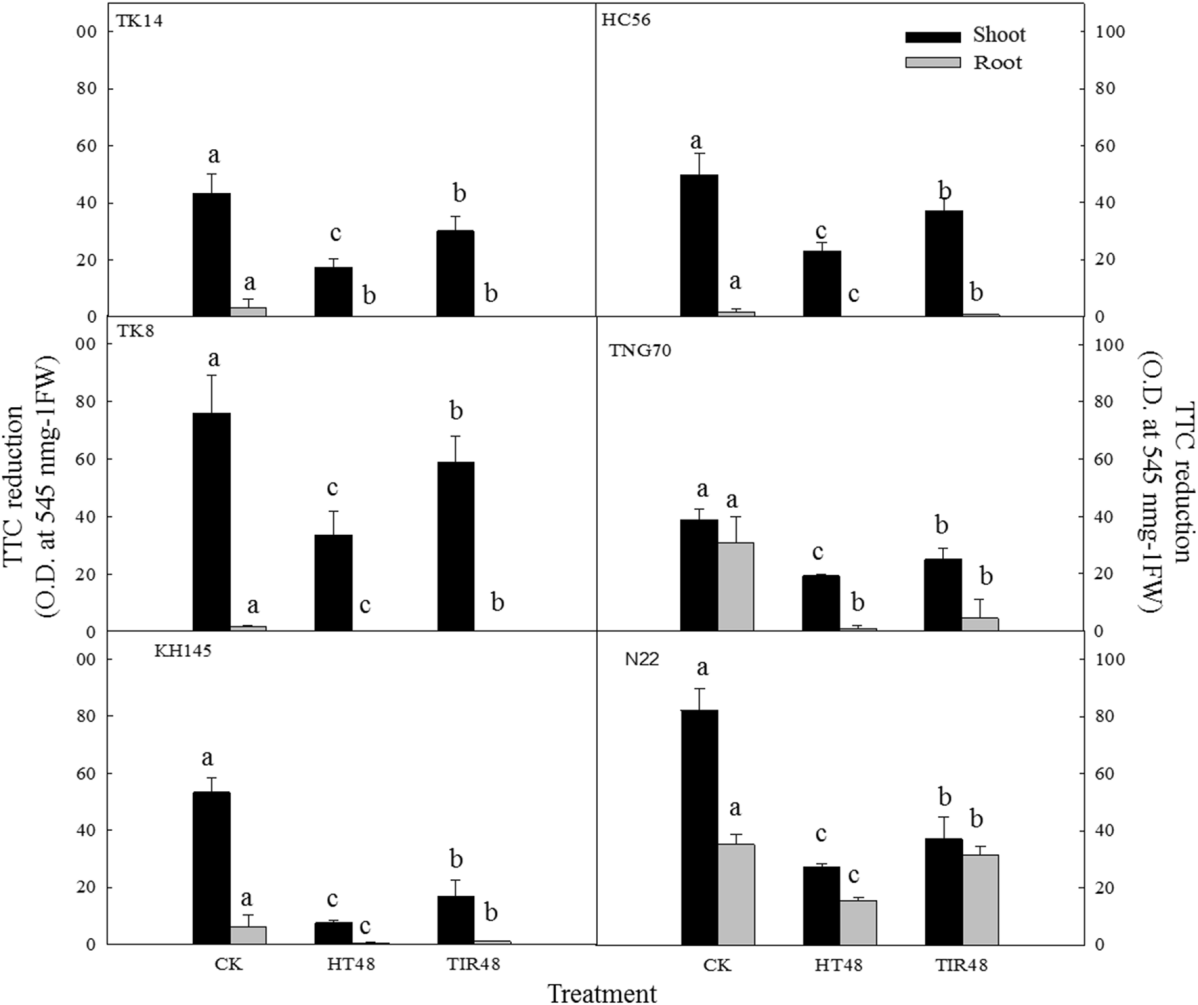
TTC reduction (O.D. at 545 nm g^−1^ FW) of the shoots and roots of the seedlings under HT48 and TIR48 (O.D./g); different letters above the same color bars indicate a significant difference at the 0.05 level

**Fig. 10:**
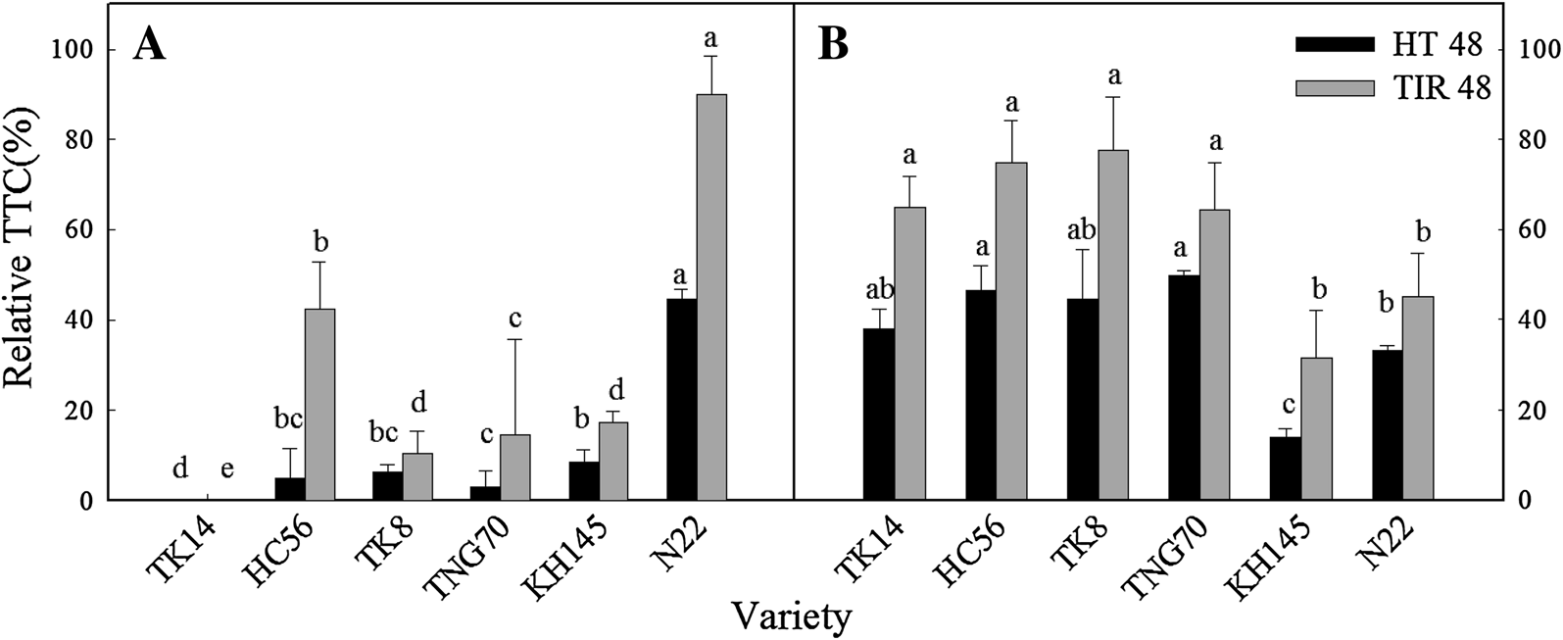
Relative TTC (%) of the roots (**A**) and shoots (**B**) of rice seedlings grown using the wrapping method under HT48 and TIR48; different letters above the same color bars indicate a significant difference at the 0.05 level

Observing the seedling tissue activity of each variety revealed that the TTC content was greater in the shoots than in the roots of every variety (Fig. 9). Comparing the shoots of each variety showed that N22 and TK8 exhibited the highest activity, whereas TNG70 and TK14 demonstrated the lowest. Of the treatment methods, the CK and HT48 treatments led to the highest and lowest activities, respectively. Calculating the relative activity of each variety revealed that all varieties exhibited significantly lower relative activity under HT48 than under TIR48 (Fig. 10). Regarding the relative activity of the shoot, the results demonstrated that the thermotolerant varieties exhibited higher cell activity (Fig. 10B). However, observing the relative activity of the roots showed that the thermosensitive variety N22 and the thermotolerant variety TK14 exhibited the highest and lowest relative activity, respectively (Fig. 10A). Therefore, under high temperatures, the root activity might be less related to the thermotolerance performance.

**Fig. 11:**
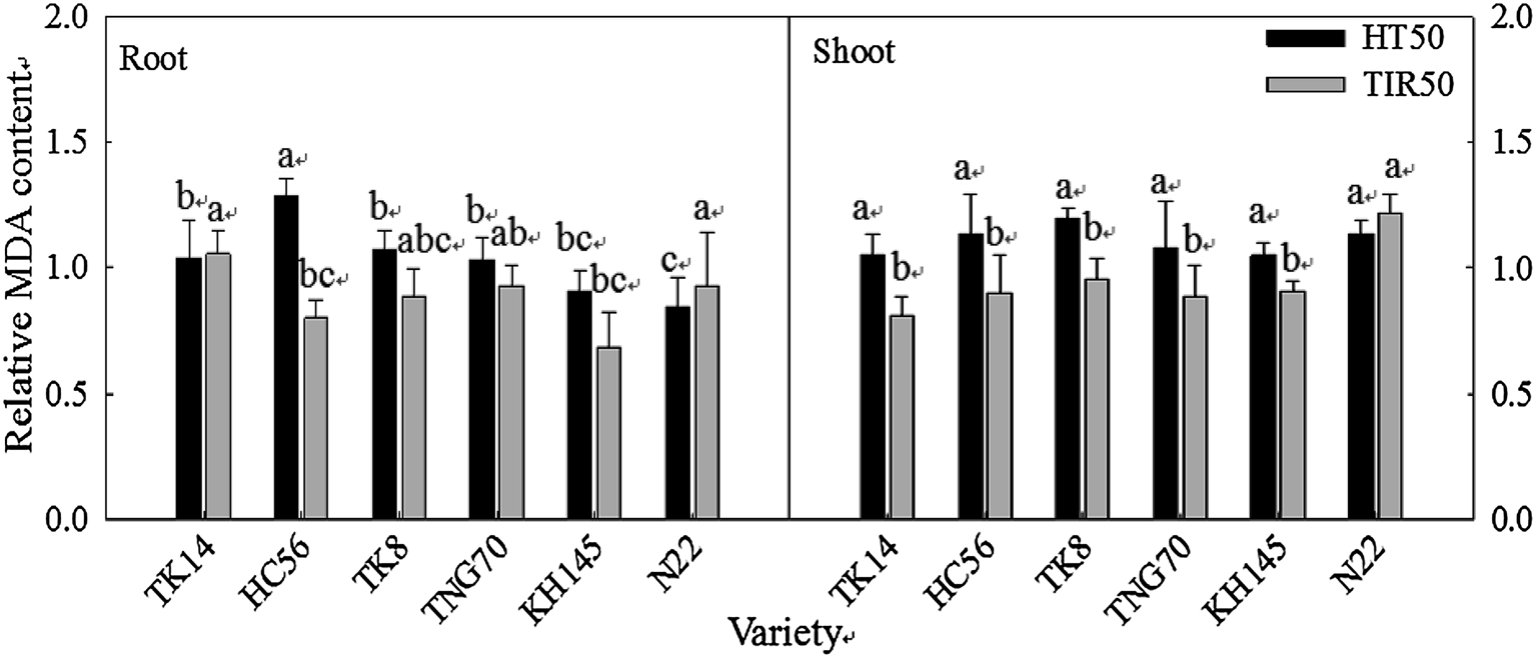
Relative MDA content in the roots and shoots of rice seedlings grown using the wrapping method under HT50 and TIR50; different letters above the same color bars indicate a significant difference at the 0.05 level

Figure 11 displays changes in the relative MDA content of the shoots and roots in the thermotolerant and thermosensitive varieties under HT50 and TIR50. After undergoing TIR50, all seedlings (excluding N22) exhibited reduced MDA content in the seedling cell membranes. Additionally, the relative MDA content in the shoots of the thermotolerant varieties was significantly lower than that in the shoots of the sensitive species N22 but was nonsignificantly different from that of KH145.

## Discussion

### Rice seedling screening method

In this study, the HT treatment was employed to screen rice seedlings with basal thermotolerance. Among the three experimental temperatures, the survival rates of the varieties under the HT45 treatment exhibited significant differences and were widely distributed. When the treatment temperature exceeded 48 °C, the plants’ survival and growth were severely inhibited; this reduced the differences in the seedling survival rate and shoot RGR between varieties. Therefore, HT45 was more adequate for evaluating the survival rate of rice seedlings because of the greater differences observed between varieties.

TIR can be used to evaluate short-term acquired thermotolerance. The treatment method in this study referenced that in Harihar et al. (2014) and Vijayalakshmi et al. (2015) and was aimed at providing solutions to the high air- and water-temperature stress faced by seedlings during Taiwan’s second crop seedling period. The study results indicated that the varieties subjected to the TIR48 treatment demonstrated significant differences in the shoot RGR. By contrast, significant differences were observed between the survival rates of the varieties that underwent the TIR50 treatment. After undergoing the TIR treatment, the rice seedlings’ thermotolerance was substantially improved; this finding concurred with that of Vijayalakshmi et al. (2015). Harihar et al. (2014) and Vijayalakshmi et al. (2015) employed growth and survival rates to screen plants following TIR treatment. However, the results of the present study indicated that the survival and growth rates were slightly correlated. A possible reason for this observation is that the two factors were influenced by different biological mechanisms. It is recommended to employ the shoot RGR of varieties subject to TIR48 as an indicator in future studies on screening varieties with acquired thermotolerance.

In Table 3, we also noticed that although the survival rate in most of the varieties decreased with temperature from HT45 and HT48 to HT50. However, the survival rate data of several varieties between HT48 and HT50 did not show a downward trend as expected. The reason may be that when the temperature reached the lethal critical temperature of 48 °C, the rice seedlings would be in a state of near death, but it was not easy to determine whether the plants were completely dead or still slightly viable. The determination of whether the plant died or not may be a possible cause of the survival rate data of these varieties not being as expected. In addition, the temperature difference between the HT48 and HT50 treatments was only 2 °C, and temperature errors in the temperature control circulating water bath tank for the high temperature treatment may also be one of the reasons. However, if the temperature interval was increased to compare HT45 and HT48 or HT45 and HT50, it would show a downward trend with the temperature rise. The same problem was slightly smaller on the RGR shown in Table 4. Although there were several varieties with this phenomenon, the data gap was generally small and within the experiment error. Additionally, the survival rate could only be determined by death and survival, and the RGR could show the quantity difference. Therefore, shoot RGR may be a better screening index near the critical temperature of lethality.

In this study, N22 exhibited inferior tolerance performance regardless of whether the HT or TIR treatments were applied. This result differed from that of Vijayalakshmi et al. (2015), who indicated that N22 TIR-screened plants that had been grown in a petri dish and undergone treatment in a growth chamber exhibited high thermotolerance. The main reason may be that the two studies adopted different treatment conditions. The water-bath method employed in the present study subjected the rice seedlings to high temperature and hypoxia conditions, causing harsher stress environments. Under such a stress environment, genes that are involved in related mechanisms in a plant’s growth might differ. Yeh et al. (2012) compared HT treatments in water baths with those administered in ovens and growth chambers; their results indicated that because water exhibits greater thermal conductivity than air, water-bath treatments are more efficient at delivering heat stress temperatures than treatments using ovens or growth chambers. This was assumed to be why the fatal temperature of the seedlings observed in the present study (48 °C) was slightly lower than that in other studies employing growth chambers (52–54 °C). However, the stress treatments adopted in this study may be helpful in screening varieties that exhibit outstanding tolerance in both high temperatures and high humidity.

The TIR treatment method employed in this study was similar to that used by Harihar et al. (2014) and Vijayalakshmi et al. (2015). Despite the present study adopting slightly different fatal temperatures and recommended screening temperatures than the abovementioned two relevant studies, the rice seedlings exhibited significantly different enhanced survival and growth rates after temperature induction (Tables 1, 3 and 4); furthermore, some varieties exhibited substantial thermotolerance improvements, whereas other varieties’ extent of improvement was low. Accordingly, the temperature induction approach could be used for not only variety screening but also for seedling nursery processes that require increasing the seedling thermotolerance. TIR has been applied as a screening method to select thermotolerant varieties from numerous crops with the potential to acquire thermotolerance ability after temperature induction. The selected high-thermotolerance varieties can be grown into fields to select other favorable traits in future generations; this can serve as a nondestructive and high-throughput screening method (Harihar et al. 2014) that can be applied to selecting breed offspring in hybridization groups during the breeding process.

### Thermotolerance evaluation results of Taiwanese subtropical rice seedlings

This study adopted multiple HT (i.e., HT45, HT48, and HT50) and post-induction high temperature treatments (TIR48 and TIR50) to evaluate and screen 27 varieties. These varieties exhibited significantly different thermotolerances under various treatments, which were then used to separately identify several thermotolerant and thermosensitive varieties (Table 5). In the future, depending on breeding needs, these varieties with proven tolerance could be used for further heat-tolerant breeding with enhanced heat tolerance ability. This study combined HT and TIR screening to identify TK14, HC56, TT30, TK8, TNG70, and HL21 as thermotolerant varieties and N22, KH145, and Koshiibuki as thermosensitive varieties. Furthermore, because HC56, TK14, and TT30 demonstrated outstanding thermotolerance under the HT and TIR treatments, they are good candidates for future breeding processes.

By employing the shoot RGR under the TIR48 treatment as an indicator, this study compared local Taiwan varieties with Koshihikari, the main cultivar in Japan, and demonstrated that 13 Taiwan varieties exhibited higher thermotolerance than Koshihikari. Additionally, under the TIR50 and HT45 treatments, 16 and 9 Taiwan varieties demonstrated greater survival rates than Koshihikari, respectively. In sum, because Taiwan is located in a subtropical zone, it has significantly higher temperatures than Japan. There are many Taiwan varieties that exhibit greater thermotolerance in the seedling stage than in Japan, most of which are Japonica-type varieties. These local Taiwan germplasms exhibit greater thermotolerance in the seedling stage and could be a valuable resource for future breeding and utilization in nearby countries or regions.

### Evaluation and physical property analysis of the screened thermotolerant and thermosensitive varieties

Because the thermotolerant varieties were verified to exhibit similar results through both potted and wrapped cultivation under the TIR48 treatment (Fig. 8), the paper-wrapped water bath method could be employed as an initial screening approach for a large number of varieties.

The continuity of crop growth is mainly determined by the thermostability of the cell membrane (CMS) under heat stress; therefore, thermotolerant varieties with high CMS and low MDA contents exhibit less cell membrane structural damage (Liu et al. 2013, 2017). After this study conducted the TIR treatment, except for the thermosensitive variety N22, all other varieties were shown to exhibit a significant decrease in MDA.

TTC has a high reduction potential and can enter the electron transport chain in mitochondrial respiration. The colorless TCC reacts with dehydrogenase and is reduced to an insoluble red precipitate. The higher the cell activity, the closer the cells are to a dark red color. TTC can be used as a cell activity measurement method to evaluate the survival rate of plant cells and tissues under HTs (Towil and Mazur 1974). According to the TTC analysis, the thermotolerant varieties exhibited greater shoot relative activity, whereas the thermosensitive varieties exhibited greater root relative activity. Because the root activity of each variety differed substantially before heat treatment, the converted relative TTC value could not be used for comparing cell activity. Therefore, the CMS, TTC, and MDA of the shoots could be used instead as the thermotolerance evaluation indicators of rice seedlings during early germination. Fokar et al. (1998) pointed out that the CMS and TTC analysis of heat-acclimated wheat seedling leaves can be used as a heat-resistance screening index; here, despite a high correlation between CMS and TTC, seedling leaf CMS demonstrated greater predictability of the thermotolerance of crop yield at the harvest stage than TTC. Prasad et al. (2006) further analyzed leaf membrane stability and spikelet fertility at the harvest stage, and indicated that no significant relationship exists and that the CMS factor can only serve as partial indication for identifying sensitive species.

### Suggestions for future breeding strategies

This study analyzed the phylogenetic dendrogram of 27 varieties (Fig. 2), the results of which showed that thermotolerant varieties were scattered across clusters instead of being concentrated in specific clusters. This indicated that the possible thermotolerance genes were distributed in different clusters. Because thermotolerance mechanisms are enabled by comprehensive gene expressions that are relatively complex, such mechanisms involve numerous thermotolerant genes. Thus, future breeding practices should select thermotolerant varieties from different phylogenetic clusters as parents to stack thermotolerance-related genes. In addition, the year a variety was released did not correlate with its thermotolerance (Fig. 3), indicating that the breeding environment under a gradually warming climate in recent years has not naturally resulted in the improving thermotolerance of newly-bred varieties. Possible reasons for this include that thermotolerance has not been prioritized as a selection trait during the breeding process. Therefore, the selected varieties featured high-production, early maturity, high-quality, and resistance to pests and diseases traits, but lacked thermotolerance traits.

At present, the varieties verified as exhibiting outstanding thermotolerance in spikelet fertility under HTs include N22, Koshiibuki, and Giza 178 (Hoshi et al. 2001). In the present study, the three varieties exhibited excellent relative germination rates but poor seedling thermotolerance performances. Because HT impedes the germination of all rice species, substantial performance differences can be observed in the germination and seedling growth rates of different varieties when HT is applied. Additionally, the germination environment temperature should be lower than 42 °C to avoid delaying germination or completely inhibiting the germination process; however, the main germination rate still depends on the variety (Ali et al. 2013). In addition, the rice seedling survival rate, shoot RGR, and germination rate exhibited a low correlation, mainly because seed germination requires the seed embryo to provide nutrition and heat stress influences amylase activity, which affects the embryo’s nutrition supply and even causes embryo death (Essemine et al. 2010). During the initial seedling stage, in addition to requiring the embryo to provide nutrients, the plant must uphold the interaction between root and shoot to maintain growth. Under heat stress, the plant faces crucial water losses (Al-Busaidi et al. 2012), which influence the shoot development as well as the quantity and diameter of roots grown during the germination stage (Porter and Gawith 1999). During different growth stages, rice exhibits different thermotolerance responses. This study assumed the reason to be that the thermotolerance responses of different growth stages are induced by different mechanisms and genes. Therefore, the thermotolerance properties exhibited in a growth stage are not directly related to those in other growth stages. This means that screening for the thermotolerance traits associated with fertility should be conducted during the fertility stage, and that screening for the thermotolerance traits associated with seedling growth and survival should be conducted during the seedling stage.

The screen tools, thermotolerance varieties and discovery of this study could be valuable resources and a reference for countries that grow Japonica type rice to apply when breeding thermotolerant varieties in the future.

## Conclusions

In this study, the HT and TIR screening tools were established to screen the heat tolerance of Taiwan rice varieties at seedling stage. Several varieties (i.e., TK14, HC56, TT30, TNG70, and TK8) obtained through the screenings exhibited outstanding thermotolerance. Our results suggest that progressive temperature induction treatment could substantially improve the rice seedlings’ thermotolerance under high temperatures stress. In addition, the CMS, TTC, and MDA of the shoots could also be used as the thermotolerance evaluation indicators of rice seedlings during early germination. On the other hand, thermotolerance during different growth stages (i.e., the germination, seedling, and grain maturation stages) exhibited low correlations. In the future, thermotolerance breeding for specific growth periods should be conducted in the target growth stage. The screen tools, thermotolerance varieties and discovery of this study could be valuable resources and references for future breeding and utilization in nearby countries or regions that grow Japonica type rice.

## Data Availability

Not applicable.

## Abbreviations

TIR: temperature induction response technique
BT: basal thermotolerance
SAT: short-term thermotolerance
LAT: long-term thermotolerance
TMHT: thermotolerance to moderately high temperatures
IPCC: Intergovernmental Panel on Climate Change
shoot RGR: shoot relative growth rate
CK: control
HT: high temperature treatment
RI: relative injury
TTC: 2,3,5-triphenyl-tetrazolium chloride
MDA: malondialdehyde content
CMS: cell membrane stability

## References

[CR1] Al-Busaidi A, Ahmed M, Chikara J (2012). The impact of heat and water stress conditions on the growth of the biofuel plant *Jatropha curcas*. Int J Environ Stud.

[CR2] Ali K, Azhar A, Galani S (2013). Response of rice (*Oryza sativa* L.) under elevated temperature at early growth stage: physiological markers. Russ J Agric Socio Econ Sci.

[CR3] Babu DV, Sudhakar P, Reddy YSK (2013). Screening of thermotolerant ragi genotypes at seedling stage using TIR technique. Bioscan.

[CR4] Baker JT, Allen LH, Boote KJ (1992). Temperature effects on rice at elevated CO_2_ concentration. J Exp Bot.

[CR5] Chen H, Lin MS, Hwu KK (2008). A database of Taiwan rice pedigrees. Crop Environ Bioinform.

[CR6] Chou C, Chen WT, Lo MH, Hsu HH, Hong CC, Tsou CH, Lu MM, Hung CW, Chen CT, Cheng CZ (2017) Climate change in Taiwan 2017: Scientific Report 1-The Physical Science Basis. https://tccip.ncdr.nat.gov.tw/v2/upload/book/20180410112426.pdf

[CR7] Cox TS, Murphy JP, Rodgers DM (1986). Changes in genetic diversity in the red winter wheat regions of the United States. Proc Natl Acad Sci USA.

[CR8] Essemine J, Amnar S, Bouzid S (2010). Importance of heat stress on germination and growth in higher plants: biochemical and molecular repercussions and mechanism of tolerance. J Biol Sci.

[CR9] Fokar M, Henry TN, Blum A (1998). Heat tolerance in spring wheat: I. Estimating cellular thermotolerance and its heritability. Euphytica.

[CR10] Fukushima A, Ohta H, Kaji R, Tsuda N (2015). Effects of hot water disinfection and cold water seed soaking on germination in feed rice varieties of Tohoku region. Jpn J Crop Sci.

[CR11] Harihar S, Srividhya S, Vijayalakshmi C, Boominathan P (2014). Temperature induction response technique—a physiological approach to identify thermotolerant genotypes in rice. Int J Agric Sci.

[CR12] HoShi T, Abe S, Kasaneyama H, Kobayashi K, Hirao K, Matsui T, Tamura T, Asai Y, Nakajima K, Kanayama H, Sasaki Y, Abe N, Azuma S, Kondo T, Ishizaki K, Higuchi K, Ozeki M, Harada A (2001). A new rice cultivar “Koshiibuki” with high grain quality and excellent taste. Hokuriku Crop Sci.

[CR13] Hsu PY, Yeh DM (2004). Heat tolerance in English ivy as measured by an electrolyte leakage technique. J Hortic Sci Biotechnol.

[CR14] IPCC (2007) Climate change 2007: the physical basis. Summary for Policy Makers. http://www.picc.ch

[CR15] Field CB, Barros V, Stocker TF, Qin D, Dokken DJ, Ebi KL, Mastrandrea MD, Mach KJ, Plattner GK, Allen SK, Tignor M, Midgley PM, IPCC (2012). Summary for policymakers. Managing the risks of extreme events and disasters to advance climate change adaptation: a Special Report of Working Groups I and II of the Intergovernmental Panel on Climate Change.

[CR16] Kao CH (2005). Laboratory manual for physiological studies of plants.

[CR17] Kempthorne O (1969). An introduction to genetic statistics.

[CR18] Kheir MS, Sheshshayee T, Prasad G, Udayakumar M (2012). Temperature induction response as a screening technique for selecting high temperature-tolerant cotton lines. J Cotton Sci.

[CR19] Kobata T, Uemuki N, Inamura T, Kagata H (2004). Shortage of assimilate supply to grain increases the proportion of milky white rice kernels under high temperatures. Jpn J Crop Sci.

[CR21] Kumar G, Krishnaprasad BT, Savitha M, Gopalakrishna R, Mukhopadhyay K, Ramamohan G, Udayakumar M (1999). Enhanced expression of heat shock protein in thermotolerant lines of sunflower and their progenies selected on the basis of temperature induction response (TIR). Theor Appl Genet.

[CR22] Lin AC, Chen JS (1976). Comparison of tillering characteristics of rice in the first and second phases. Sci Dev.

[CR23] Lin MS, Wu CC (1994). Program for estimating relative genetic contribution and coefficient of parentage. J Hered.

[CR24] Liu QH, Wu X, Li T, Ma JQ, Zhou XB (2013). Effects of elevated air temperature on physiological characteristics of flag leaves and grain yield in rice. Chilean J Agric Res.

[CR25] Liu Shuo, Waqas Muhammad Ahmed, Wang Song-he, Xiong Xiang-yang, Wan Yun-fan (2017). Effects of increased levels of atmospheric CO2 and high temperatures on rice growth and quality. PLOS ONE.

[CR26] Lur HS (2009) Effects of high temperature on yield and grain quality of rice in Taiwan. In: MARCO symposium. challenges for agro-environmental research in monsoon Asia; National Institute for Agro-Environmental Science, Japan. http://www.niaes.affrc.go.jp/marco/marco2009/english/W2-06Huu-ShengLurP.pdf

[CR27] Nagata K, Takita T, Yoshinaga S, Terashima K, Fukuda A (2004). Effect of air temperature during the early grain filling stage on grain fissuring in rice. Jpn J Crop Sci.

[CR28] Oh-e I, Saitoh K, Kuroda T (2007). Effects of high temperature on growth, yield and dry-matter production of rice grown in the paddy field. Plant Prod Sci.

[CR29] Peng S, Huang J, Sheehy JE, Laza RC, Visperas RM, Zhong X, Centeno GS, Khush GS, Cassman KG (2004). Rice yields decline with higher night temperature from global warming. Proc Natl Acad Sci USA.

[CR30] Permana H, Murata K, Kashiwagi M, Yamada T, Kanekatsu M (2017). Screening of Japanese rice cultivars for seeds with heat stress tolerance under hot water disinfection method. Asian J Plant Sci.

[CR32] Porter JR, Gawith M (1999). Temperature and the growth and development of wheat: a review. Eur J Agron.

[CR33] Prasad PVV, Boote KJ, Allen LH, Sheehy JE, Thomas JMG (2006). Species, ecotype and cultivar differences in spikelet fertility and harvest index of rice in response to high temperature stress. Field Crops Res.

[CR34] Satake T, Matsuo T, Kumazawa K, Ishii R, Ishihara K, Hirata H (1995). High temperature injury. Science of the rice plant, vol. 2: physiology.

[CR35] Srikanthbabu V, Ganeshkumar B, Krishnaprasad T, Gopalakrishna R, Savitha M, Udayakumar M (2002). Identification of pea genotypes with enhanced thermotolerance using temperature induction response technique (TIR). J Plant Physiol.

[CR36] Tenorio FA, Ye C, Redona E, Sierra S, Laza M, Argayoso MA (2013). Screening rice genetic resources for heat tolerance. SABRAO J Breed Genet.

[CR37] Towil LE, Mazur P (1974). Studies on the reduction of 2,3,5-triphenyl tetrazolium chloride as a viability assay for plant tissue culture. Can J Bot.

[CR38] Vijayalakshmi D, Srividhya S, Vivitha P, Raveendran M (2015). Temperature induction response (TIR) as a rapid screening protocol to dissect the genetic variability in acquired thermotolerance in rice and to identify novel donors for high temperature stress tolerance. Ind J Plant Physiol.

[CR39] Wu WC (2008) Genetic base and diversity of Japonica rice varieties of Taiwan based on pedigree analysis. Master thesis, National Chung Hsing University, Taiwan

[CR40] Wu WC, Lin MS (2008). Pedigree analysis of rice varieties of Taiwan: I. Relationships among Japanese introductions. Crop Environ Bioinform.

[CR41] Yang JL, Wu ST, Thseng FS (1997). Variation of emergence ability of rice using direct-seeding method.

[CR42] Yeh CH, Kaplinsky NJ, Hu C, Charng YY (2012). Some like it hot, some like it warm: phenotyping to explore thermotolerance diversity. Plant Sci.

